# Accelerated Senescence and Enhanced Disease Resistance in Hybrid Chlorosis Lines Derived from Interspecific Crosses between Tetraploid Wheat and *Aegilops tauschii*

**DOI:** 10.1371/journal.pone.0121583

**Published:** 2015-03-25

**Authors:** Hiroki Nakano, Nobuyuki Mizuno, Yukio Tosa, Kentaro Yoshida, Pyoyun Park, Shigeo Takumi

**Affiliations:** 1 Laboratory of Plant Genetics, Graduate School of Agricultural Science, Kobe University, Kobe, Japan; 2 Laboratory of Plant Pathology, Graduate School of Agricultural Science, Kobe University, Kobe, Japan; 3 Laboratory of Stress Cytology, Graduate School of Agricultural Science, Kobe University, Kobe, Japan; Nanjing Agricultural University, CHINA

## Abstract

Hybrid chlorosis, a type of hybrid incompatibility, has frequently been reported in inter- and intraspecific crosses of allopolyploid wheat. In a previous study, we reported some types of growth abnormalities such as hybrid necrosis and observed hybrid chlorosis with mild or severe abnormalities in wheat triploids obtained in crosses between tetraploid wheat cultivar Langdon and four *Ae*. *tauschii* accessions and in their derived synthetic hexaploids. However, the molecular mechanisms underlying hybrid chlorosis are not well understood. Here, we compared cytology and gene expression in leaves to characterize the abnormal growth in wheat synthetics showing mild and severe chlorosis. In addition, we compared disease resistance to wheat blast fungus. In total 55 and 105 genes related to carbohydrate metabolism and 53 and 89 genes for defense responses were markedly up-regulated in the mild and severe chlorosis lines, respectively. Abnormal chloroplasts formed in the mesophyll cells before the leaves yellowed in the hybrid chlorosis lines. The plants with mild chlorosis showed increased resistance to wheat blast and powdery mildew fungi, although significant differences only in two, third internode length and maturation time, out of the examined agricultural traits were found between the wild type and plants showing mild chlorosis. These observations suggest that senescence might be accelerated in hybrid chlorosis lines of wheat synthetics. Moreover, in wheat synthetics showing mild chlorosis, the negative effects on biomass can be minimized, and they may show substantial fitness under pathogen-polluted conditions.

## Introduction

Allopolyploid speciation, which is one of the major evolutionary processes in higher plants [1,2], is achieved through various processes including interspecific hybrid formation and endoreduplication. In interspecific crosses, the hybrid plants sometimes break down, a phenomenon called hybrid incompatibility. Hybrid incompatibility inhibits allopolyploid speciation. Common wheat (*Triticum aestivum* L., 2n = 6x = 42, genome constitution AABBDD) is an allohexaploid species derived through allohexaploidization between cultivated tetraploid wheat (*Triticum turgidum* L., 2n = 4x = 28, AABB genome) and wild diploid progenitor *Aegilops tauschii* Coss. (2n = 2x = 14, DD genome) [3]. Hexaploid wheat plants with the AABBDD genome can be obtained through artificial hybrids (2n = 3x = 21, ABD genome) between tetraploid wheat and *Ae*. *tauschii*, called synthetic hexaploid wheat [4]. Growth incompatibilities were first reported half a century ago in ABD triploids [5–7]. Recently, the tetraploid wheat cultivar Langdon (Ldn) was found to be an efficient AB genome parent for the production of synthetic hexaploid wheat [8]. Numerous synthetic wheat hexaploids have been produced from ABD hybrids between Ldn and various *Ae*. *tauschii* accessions [9], whereas several types of hybrid growth abnormalities were observed in many cross combinations [10,11]. The incompatibilities between the wheat AB and D genomes include hybrid chlorosis, severe growth abortion, and two types of hybrid necrosis (type II and type III necrosis), which function as postzygotic reproductive barriers preventing the production of synthetic hexaploid wheat [11]. Cell death occurs gradually beginning with older tissues in hybrid lines showing type III necrosis, whereas type II necrosis lines show a necrotic phenotype under low temperature conditions [11].

Most hybrid incompatibilities are caused by interaction of at least two loci or alleles, a basis of the Dobzhansky-Muller model [12]. Hybrid necrosis is generally triggered by autoimmune response, and the causal genes encode disease resistance-related proteins in higher plants such as *Arabidopsis* and lettuce [13–16]. The up-regulation of defense-related genes such as pathogen-related genes is observed in plants exhibiting hybrid weakness as well as hybrid necrosis, and the causal genes for hybrid weakness are also disease resistance genes in rice [17,18]. In ABD triploids, the programmed cell death of severe growth abortion and two types of hybrid necrosis, type II and III necrosis, is accompanied by a significant increase in expression of defense-related genes [11,19,20]. Up to now, no gene expression profiles of ABD hybrid plants showing hybrid chlorosis have been reported. Leaves of the hybrid plants exhibiting chlorosis gradually turn yellowish with age. Four *Ae*. *tauschii* accessions reportedly induce hybrid chlorosis in ABD triploids crossed with Ldn [11]. The chlorosis phenotype is different from that of the hybrid chlorosis observed in interspecific crosses between a few accessions of cultivated emmer wheat (*T*. *turgidum* ssp. *dicoccon* (Schrank) Thell., AABB genome) and many accessions of cultivated and wild timopheevii wheat (*Triticum timopheevii* Zhuk., AAGG genome); hybrid chlorosis in tetraploid wheat is lethal [21]. Hybrid chlorosis between *T*. *turgidum* and *T*. *timopheevii* is controlled by two dominant complementary genes, *Cs1* on chromosome 5A and *Cs2* on chromosome 4G [22]. In tetraploid and hexaploid wheat, interaction between *Ch1* on 2A and *Ch2* on 3D induces hybrid chlorosis with lethality [23–25]. In addition, three chlorophyll-related abnormalities, striato-virescence, delayed virescence, and albino, are triggered by complementary gene interactions on chromosomes 2A, 3A, or 2B in tetraploid wheat [26]. Wheat plants showing striato-virescence and delayed virescence are viable and fertile, although albinos are not.

Intraspecific hybrids with severe growth defects show enhanced disease resistance, and in *Arabidopsis*, the enhanced resistance is related to activation of the salicylic acid (SA) stress signaling pathway [14]. Most plant lesion mimic mutants with either necrotic or chlorotic lesions have activated defense responses and show increased resistance to virulent pathogens compared with wild-type plants [27]. Reduced growth in the increased-resistance plants is assumed to be due to the high cost for maintenance of defense response-related pathways [14,28,29]. In the ABD triploids with growth abnormalities, phenotypic effects of hybrid chlorosis seem milder than hybrid necrosis and severe growth abortion [10,11]. The objectives of this study were: 1) to clarify transcriptional and physiological characteristics of hybrid chlorosis of the ABD triploids, 2) to map one of the causal genes for hybrid chlorosis on the wheat D-genome chromosome, and 3) to evaluate the level of disease resistance. Based on these results, we aimed to elucidate the mechanism inducing hybrid chlorosis, and discussed the relationship between growth defects due to hybrid incompatibility and enhanced disease resistance in ABD triploid wheat.

## Results

### Phenotype of hybrid chlorosis in triploid wheat hybrids and wheat synthetics

**Fig 1:**
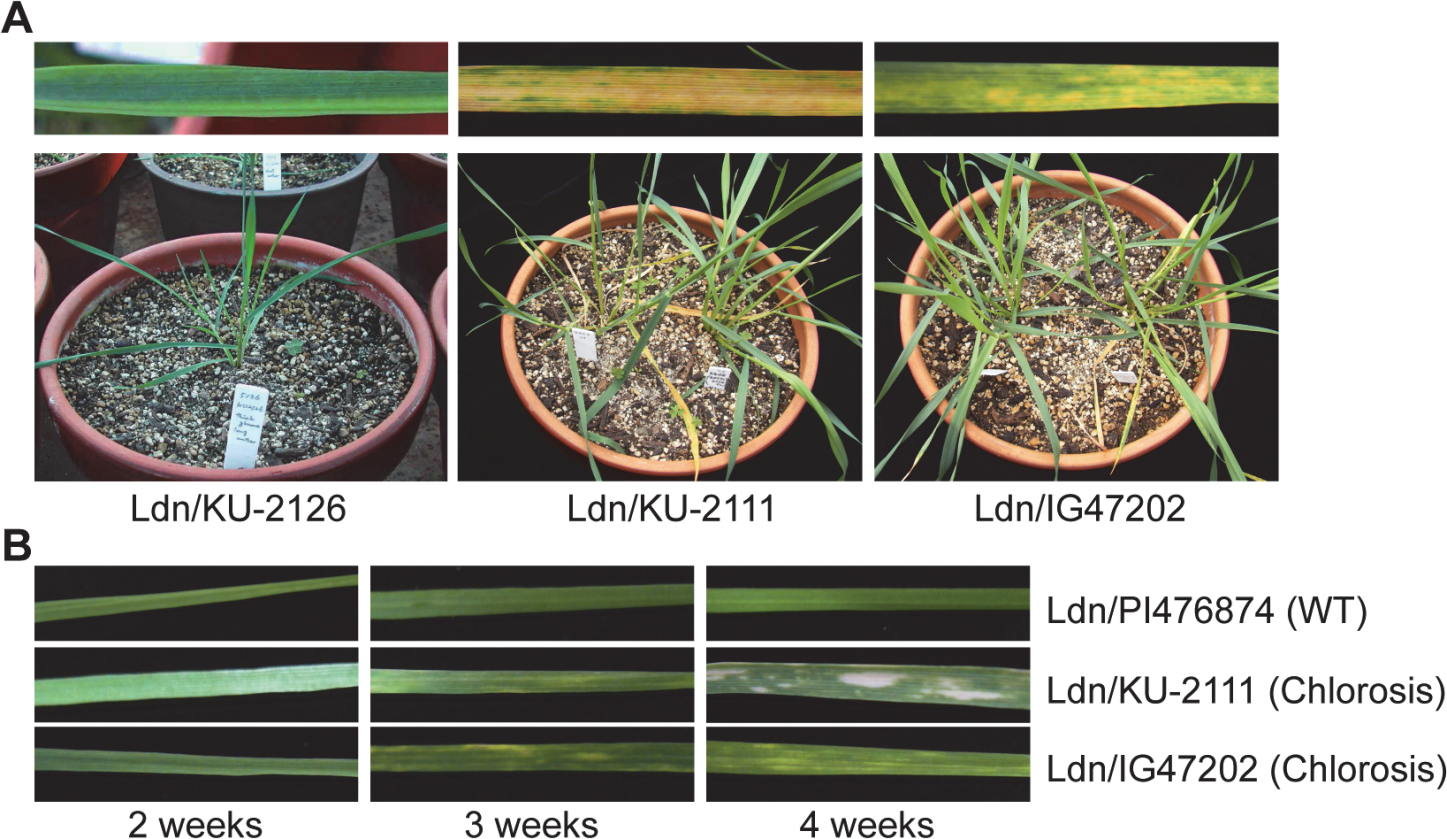
Synthetic hexaploid wheat plants exhibiting hybrid chlorosis. (A) Phenotypes of the normal growth (WT, Ldn/KU-2126), severe chlorosis (Ldn/KU-2111) and mild chlorosis (Ldn/IG47202) lines. The plants with mild chlorosis were grown for two months after sowing and showed lighter yellowish symptoms than those with severe chlorosis. (B) The first leaf phenotypes of plants with WT and with hybrid chlorosis. Each plant was grown at 23°C for 2 to 4 weeks.

Triploid plants between Ldn and *Ae*. *tauschii* that showed hybrid chlorosis were fertile, and produced selfed seeds, from which synthetic hexaploids with 42 chromosomes germinated. Two synthetic hexaploid lines, Ldn/KU-2111 and Ldn/KU-20-1, showed an identical chlorosis phenotype, whereas the chlorosis phenotype of another Ldn/IG47202 hexaploid line was milder (Fig. 1A). In the two former chlorosis lines (severe chlorosis), yellowish lesions or sectors appeared on the leaf surface gradually starting with older tissues, and then the leaves turned brown. The yellowish sectors appeared similarly, in an age-dependent manner, and the color change to brown was not observed until senescence began in the distal part of leaves in Ldn/IG47202 (mild chlorosis) (Fig. 1B). The phenotype of these hybrid chlorosis lines was not lethal, and selfed seed progeny could be obtained.

### Chromosomal location of the causal gene for hybrid chlorosis

The severe chlorosis line Ldn/KU-2111 was crossed with the mild chlorosis line Ldn/IG47202 for allelism test, and 156 F_2_ plants obtained from the cross were grown in the 2011–2012 season. The F_1_ plants seemed to show the severe chlorosis phenotype and no F_2_ progeny without any chlorosis symptoms was observed, indicating that the causal gene for severe chlorosis was closely linked or overlapped with that for mild chlorosis in the D genome.

To assign the D-genome causal gene for severe chlorosis, 96 F_2_ plants from a cross of Ldn/PI476874, showing normal growth phenotype without any chlorosis symptoms (wild type, WT), and Ldn/KU-2111 were used as a mapping population. In the F_2_ mapping population, 70 plants with chlorosis symptoms and 26 WT plants were observed. The chlorosis-inducing allele was dominant, and the segregation ratio statistically fitted a 3:1 ratio (χ^2^ value = 0.637, *P* > 0.05), which was consistent with Mendelian segregation of alleles of a single gene.

**Fig 2:**
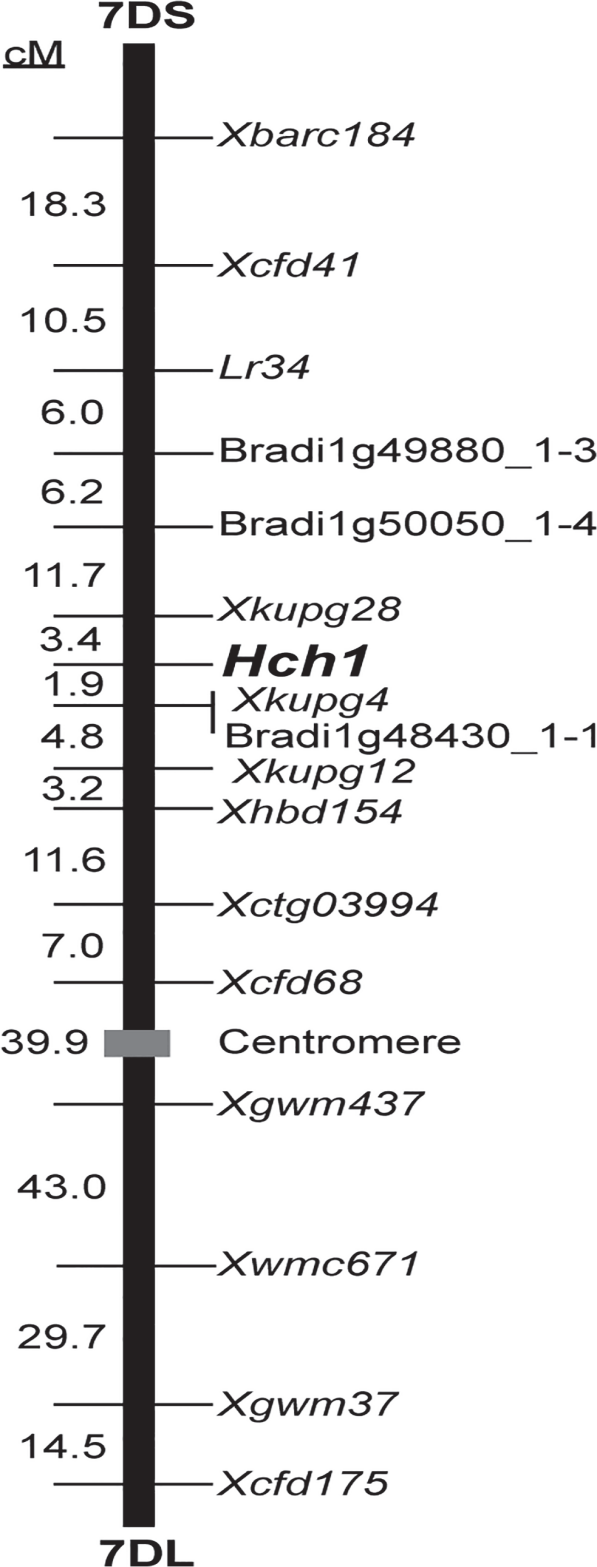
Genetic mapping of *Hch1* to the short arm of chromosome 7D. Genetic distances (cM) are shown on the left and markers on the right.

A single locus that controlled hybrid chlorosis in *Ae*. *tauschii* was assigned to the short arm of chromosome 7D in the Ldn/PI476874//Ldn/KU-2111 population (Fig. 2). The locus related to induction of chlorosis was mapped together with 11 simple sequence repeat (SSR) markers, 4 cleaved amplified polymorphic sequence (CAPS) markers, and *Lr34*. The four CAPS and three SSR markers were respectively developed based on reference to single nucleotide polymorphism (SNP) data for *Ae*. *tauschii* and survey sequence data for 7DS [30,31]. The hybrid chlorosis-related locus, named *Hybrid chlorosis1* (*Hch1*), was within a 5.3 cM interval between the two linked SSR marker loci *Xkupg4* and *Xkupg28* (Fig. 2).

### TEM observation of leaf mesophyll cells

**Fig 3:**
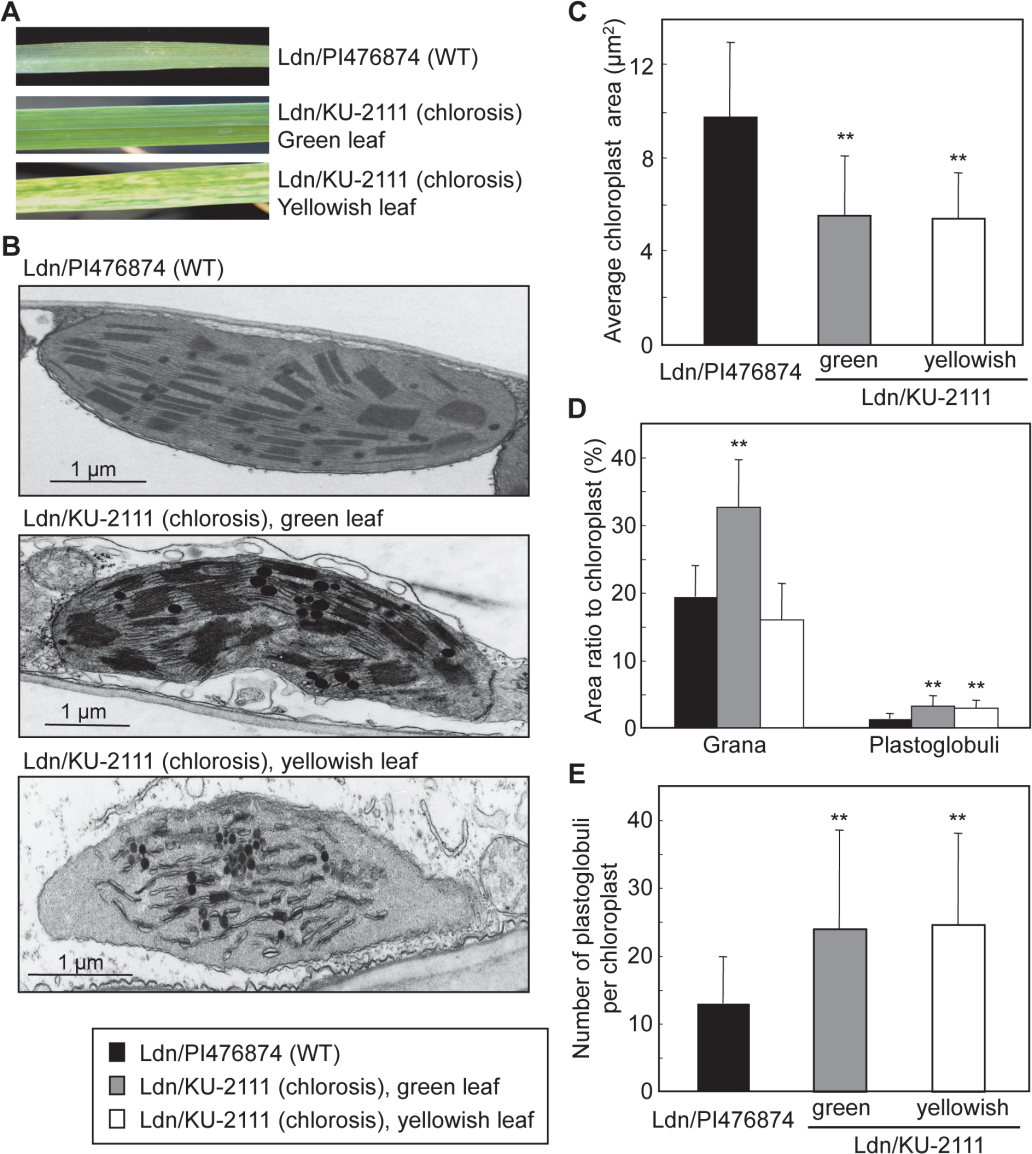
Comparative TEM ultrastructure of chloroplast structures in the mesophyll cells of the WT and chlorosis lines. (A) Leaf phenotypes in the WT and severe chlorosis lines. In the chlorosis line, two types of leaves were represented, before and after appearance of chlorosis. (B) Chloroplasts in synthetic hexaploid wheat lines showing WT appearance and hybrid chlorosis. (C) The average area of chloroplasts in mesophyll cells of three leaves examined. (D) Ratio of the grana and plastoglobuli in chloroplasts of mesophyll cells. (E) Number of plastoglobuli. The values were expressed as means ± SD from three technical replicates, in each of which at least 25 mesophyll cells were observed. Student’s *t*-test was used to evaluate statistical significance (**P < 0.01) of differences between lines.

The organelle structures of mesophyll cells were compared for green and yellowish leaves of the Ldn/PI476874 and Ldn/KU-2111 synthetic hexaploids based on transmission electron microscopy (TEM) observation (Fig. 3A). In the cells of green leaves of the Ldn/KU-2111 plants, chloroplasts were slightly shrunken compared with those of the Ldn/PI476874 plants (Fig. 3B). The area of chloroplasts photographed at random was significantly larger in Ldn/PI476874 than in green or yellowish leaves of Ldn/KU-2111 (Fig. 3C). The stromal area of chloroplasts was less in the mesophyll cells of Ldn/KU-2111 than Ldn/PI476874. In yellowish leaves, the grana were severely collapsed (Fig. 3B). The proportion of grana to chloroplast was determined for the Ldn/PI476874 and Ldn/KU-2111 plants (Fig. 3D); the area ratio was significantly larger in green leaves of Ldn/KU-2111 than in those of Ldn/PI476874.

Plastoglobuli, globular masses of lipoprotein within chloroplasts [32], were more conspicuous in chloroplasts of leaves in Ldn/KU-2111 than in Ldn/PI476874 (Fig. 3B). In fact, the number of plastoglobuli per chloroplast was significantly higher in Ldn/KU-2111 than in Ldn/PI476874, whereas no significant difference in the number of plastoglobuli was observed between the green and yellowish leaves of Ldn/KU-2111 (Fig. 3E).

### Alteration of gene expression profiles in hybrid chlorosis

To comprehensively compare gene expression profiles among WT (Ldn/PI476874) and the two hexaploid synthetics showing hybrid chlorosis (Ldn/IG47202 and Ldn/KU-2111), transcriptome analysis was performed using a wheat-specific 38k oligo DNA microarray [33]. For hybridization, total RNA was extracted from the oldest leaves of 3-week-old seedlings grown at normal temperature (23°C). After hybridization with the RNA samples, probes showing at least a threefold difference in signal intensity compared to the WT were defined as either up- or down-regulated genes.

**Fig 4:**
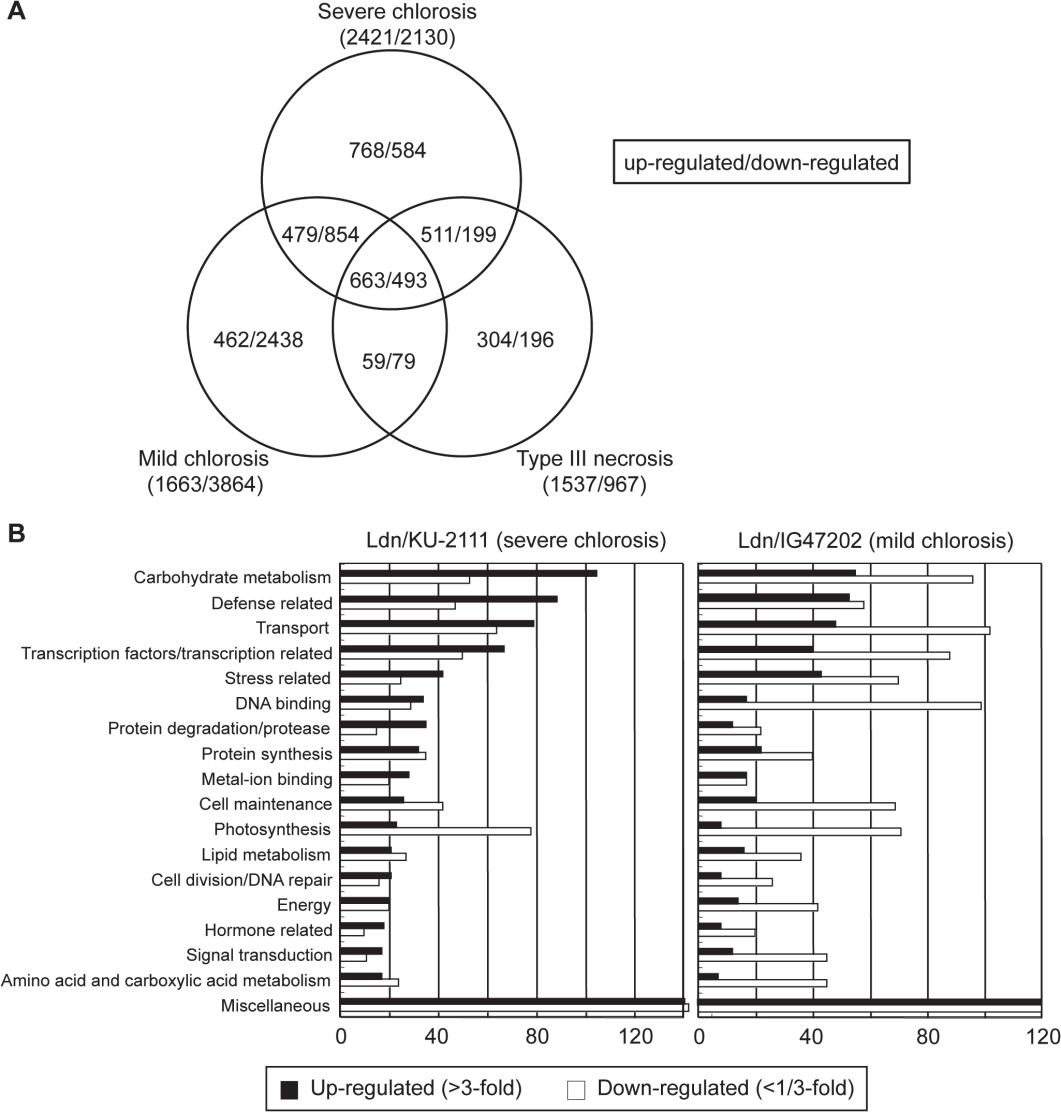
Summary of results from microarray analysis. (A) Venn diagram of genes differentially expressed between plants with severe chlorosis, mild chlorosis and type III necrosis. (B) Number of up- and down-regulated genes in leaves of the Ldn/KU-2111 and Ldn/IG47202 lines. Genes up- and down-regulated in the chlorosis line (Ldn/KU-2111) and (Ldn/IG47202) relative to WT were categorized into 18 functional groups.

Of the 37,826 probes on the wheat microarray, 1,663 (4.4%) and 3,864 (10.2%) probes were regarded as genes up- and down-regulated, respectively, in leaves of the mild chlorosis line (Ldn/IG47202) compared with the WT line (Fig. 4A). In the severe chlorosis line (Ldn/KU-2111), 2,421 (6.4%) and 2,130 (5.6%) probes were found to be up- and down-regulated, respectively.

Based on homology searches of the wheat expressed sequence tag (EST) database with probe sequences, 625 (37.6%) and 1,760 (45.5%) of up- and down-regulated genes, respectively, were categorized into a total of 17 groups based on inferred function (Fig. 4B). Of the up-regulated genes, carbohydrate metabolism-related genes were the most frequently encountered (Fig. 4B). Genes related to defense, stress, transcription factors, and transport were also abundantly expressed in the hybrid plants showing mild chlorosis. In contrast, genes related to transport, DNA binding, photosynthesis, and cell maintenance were down-regulated in these plants. Similarly, 947 (39.1%) and 786 (36.9%) genes were up- and down-regulated in the severe chlorosis line, respectively. Carbohydrate metabolism-related genes were the most frequently found to be up-regulated, whereas photosynthesis-related genes were the most dramatically down-regulated in the severe chlorosis plants.

Among the carbohydrate metabolism-related genes, beta-glucosidase, beta-1,3-glucanase precursor genes and beta-fructofuranosidase were highly up-regulated in the chlorotic plants (Tables 1 and 2). In addition, transcripts of a number of defense-related genes, including chitinase and sulfur-rich/thionin-like protein genes, accumulated abundantly in the chlorotic plants. Moreover, many WRKY-type transcription factor genes, which play important roles in immune responses, senescence, and various other processes of plants [34,35], were found among the up-regulated transcription factor genes in both chlorosis lines (Tables 3 and 4). In the severe hybrid chlorosis line, three NAC transcription factor genes including *NAM-B1*, which is associated with senescence and nutrient remobilization from leaves to developing grains [36], were up-regulated (Table 3). In both hybrid chlorosis lines, photosynthesis-related genes showed a high rate of down-regulation, including the genes for photosystem I and photosystem II proteins, chlorophyll a/b binding protein, and ribulose-1,5-bisphosphate carboxylase activase.

**Table 1:** List of the top 20 up-regulated carbohydrate metabolism genes in leaves of the severe chlorosis line Ldn/KU-2111 identified by microarray analysis.

Accession no.	Protein	*E*-value	Ratio
AAD28734	*Triticum aestivum* beta-1,3-glucanase precursor	1.00E^-98^	186.1
EMT09010	*Aegilops tauschii* 6(G)-fructosyltransferase	8.00E^-97^	125.3
ACH73192	*Triticum urartu* sucrose:fructan 6-fructosyltransferase	0	104.9
EMT19915	*Aegilops tauschii* beta-fructofuranosidase 1	3.00E^-82^	101.7
AEV76983	*Triticum aestivum* beta-glucosidase 1b	5.00E^-38^	101.5
XP_003571181	*Brachypodium distachyon* beta-fructofuranosidase 1-like	4.00E^-20^	67.7
AAR29968	*Hordeum vulgare* putative cellulose synthase catalytic subunit	5.00E^-88^	48.5
EMS54383	*Triticum urartu* trehalose-phosphate phosphatase	1.00E^-72^	48.4
2DGA_A	*Triticum aestivum* chain A, crystal structure of hexameric beta-glucosidase	0	35.9
BAE96092	*Triticum aestivum* endo-beta-1,3-glucanase	4.00E^-79^	31.8
BAE19752	*Triticum aestivum* fructan:fructan 1-fructosyltransferase	9.00E^-101^	25.4
CAG25609	*Triticum aestivum* acid beta-fructofuranosidase precursor	3.00E^-85^	24.4
EMT08337	*Aegilops tauschii* beta-glucosidase 44	1.00E^-95^	18.3
AF091802	*Aegilops tauschii* starch synthase I	4.00E^-21^	15.2
EMT15876	*Aegilops tauschii* Putative mixed-linked glucan synthase 3	4.00E^-149^	12.5
CBH32609	*Triticum aestivum* glucan endo-1,3-beta-glucosidase GII precursor	0	11.3
EMT14599	*Aegilops tauschii* beta-fructofuranosidase, soluble isoenzyme I	5.00E-^110^	10.7
BAE96092	*Triticum urartu* sucrose synthase 1	2.00E^-142^	8.8
ABR25521	*Oryza sativa* sucrose synthase metabolism	1.00E^-90^	7.9
EMT19913	*Aegilops tauschii* beta-fructofuranosidase 1	4.00E^-42^	6

**Table 2:** List of the top 20 up-regulated carbohydrate metabolism genes in leaves of the mild chlorosis line Ldn/IG47202 identified by microarray analysis.

Accession no.	Protein	*E*-value	Ratio
AEV76983	*Triticum aestivum* beta-glucosidase 1b	5.00E^-38^	471
AAD28734	*Triticum aestivum* beta-1,3-glucanase precursor	0	154
AAR29968	*Hordeum vulgare* putative cellulose synthase catalytic subunit	5.00E^-88^	113.9
AB100035	*Triticum aestivum* TaGlu1a	0	93.8
CAA77085	*Triticum aestivum* glucan endo-1,3-beta-D-glucosidase	1.00E^-98^	78.4
EMS54383	*Triticum urartu* trehalose-phosphate phosphatase	1.00E^-72^	57.9
EMT14785	*Aegilops tauschii* putative mixed-linked glucan synthase 3	2.00E^-97^	46.3
ABZ01582	*Hordeum vulgare* cellulose synthase-like CslF10	7.00E^-145^	35.2
XP_003571181	*Brachypodium distachyon* beta-fructofuranosidase 1-like	4.00E^-20^	25
XP_003577135	*Brachypodium distachyon* chalcone-flavonone isomerase-like	2.00E^-94^	24.2
ACH73192	*Triticum urartu* sucrose:fructan 6-fructosyltransferase	0	22
EMT19915	*Aegilops tauschii* beta-fructofuranosidase 1	2.00E^-23^	19.3
EU665444	*Oryza sativa* sucrose synthase metabolism	1.00E^-90^	17.3
EMS66266	*Triticum urartu* sucrose synthase 1	2.00E^-142^	17.3
GQ254772	*Triticum turgidum* starch branching enzyme IIa	7.00E^-45^	16.4
BAE19752	*Triticum aestivum* fructan:fructan 1-fructosyltransferase	9.00E^-101^	15.2
DQ286566	*Triticum aestivum* 6(G)-fructosyltransferase	1.00E-^21^	14.4
BAE96092	*Triticum aestivum* endo-beta-1,3-glucanase	4.00E^-79^	13.3
EMS64965	*Triticum urartu* 1,4-alpha-glucan-branching enzyme 2	6.00E^-10^	12.1
CAG25609	*Triticum aestivum* acid beta-fructofuranosidase precursor	4.00E^-107^	9.6

**Table 3:** The top 20 up-regulated transcription factor genes in leaves of the severe chlorosis line Ldn/KU-2111, as identified by microarray analysis.

Accession no.	Protein	*E*-value	Ratio
AB295664	*Triticum aestivum* MADS-box transcription factor	0	262.6
EU665450	*Triticum aestivum* WRKY35 transcription factor	3.00E^-48^	198.5
EU665449	*Triticum aestivum* WRKY25 transcription factor	1.00E^-74^	156.1
EF397613	*Triticum aestivum* WRKY45 transcription factor	1.00E^-114^	79.6
EF488104	*Hordeum vulgare* WRKY3 transcription factor	0	72.7
EF488105	*Hordeum vulgare* WRKY4 transcription factor	0	31.9
EU665440	*Triticum aestivum* WRKY11 transcription factor	3.00E^-53^	27.2
DQ863106	*Hordeum vulgare* WRKY22 transcription factor	2.00E^-38^	17.5
AB443456	*Triticum aestivum* WRS2 Myb transcription factor	1.00E^-167^	11.4
EF397616	*Triticum aestivum* WRKY19-b transcription factor	0	11.2
EU669662	*Triticum aestivum* WRKY32 transcription factor	0	10.6
EU730901	*Brachypodium distachyon* GRAS family transcription factor	2.00E^-23^	9.7
EU665444	*Triticum aestivum* WRKY22 transcription factor	1.00E^-126^	8.2
EU956008	*Zea mays* NAC domain-containing protein 77	1.00E^-55^	7.4
AM500853	*Hordeum vulgare* NAC transcription factor	4.00E^-19^	6.8
DQ871219	*Triticum dicoccoides* NAC transcription factor NAM-B1	1.00E^-129^	6.7
DQ286566	*Triticum aestivum* WRKY transcription factor	1.00E-^19^	5.8
EU665434	*Triticum aestivum* WRKY5 transcription factor	2.00E^-26^	5.6
AM502886	*Triticum aestivum* MADS-box transcription factor	0	5.6
DQ323512	*Triticum aestivum* WRKY28 transcription factor	3.00E^-49^	4.4

**Table 4:** The top 19 up-regulated transcription factor genes in leaves of the mild chlorosis line Ldn/IG47202, as identified by microarray analysis.

Accession no.	Protein	*E*-value	Ratio
EU665449	*Triticum aestivum* WRKY25 transcription factor	1.00E^-74^	261.8
EF488104	*Hordeum vulgare* WRKY3 transcription factor	0	172.9
EU665450	*Triticum aestivum* WRKY35 transcription factor	3.00E^-48^	142.5
AB295664	*Triticum aestivum* MADS-box protein	0	133.1
EF397613	*Triticum aestivum* WRKY45 transcription factor	1.00E^-114^	97.0
EU665440	*Triticum aestivum* WRKY11 transcription factor	3.00E^-53^	56.5
EF397616	*Triticum aestivum* WRKY19-b transcription factor	0	53.4
EF488105	*Hordeum vulgare* WRKY4 transcription factor	0	20.8
AB443456	*Triticum aestivum* WRS2 Myb transcription factor	1.00E^-167^	18.4
DQ863106	*Hordeum vulgare* WRKY22 transcription factor	2.00E^-38^	17.2
AC133007	*Oryza sativa* AP2 family transcription factor	2.00E^-26^	13.3
AP005452	*Oryza sativa* PHD finger transcription factor-like protein	3.00E^-63^	12.7
AY323206	*Hordeum vulgare* WRKY46 transcription factor	6.00E^-21^	12.0
EU665434	*Triticum aestivum* WRKY5 transcription factor	2.00E^-26^	11.2
EU730900	*Brachypodium distachyon* Myb transcription factor	3.00E^-66^	9.7
AB252151	*Triticum aestivum* Tamyb13 Myb-related protein	0	9.3
AK107483	*Oryza sativa* P-type R2R3 Myb protein	0	7.5
DQ334409	*Triticum aestivum* ERFL2a transcription factor	1.00E^-156^	7.2
EU665435	*Triticum aestivum* WRKY6 transcription factor	1.00E^-146^	7.1

### Comparison of gene expression profiles between wheat synthetics displaying chlorosis and necrosis

**Fig 5:**
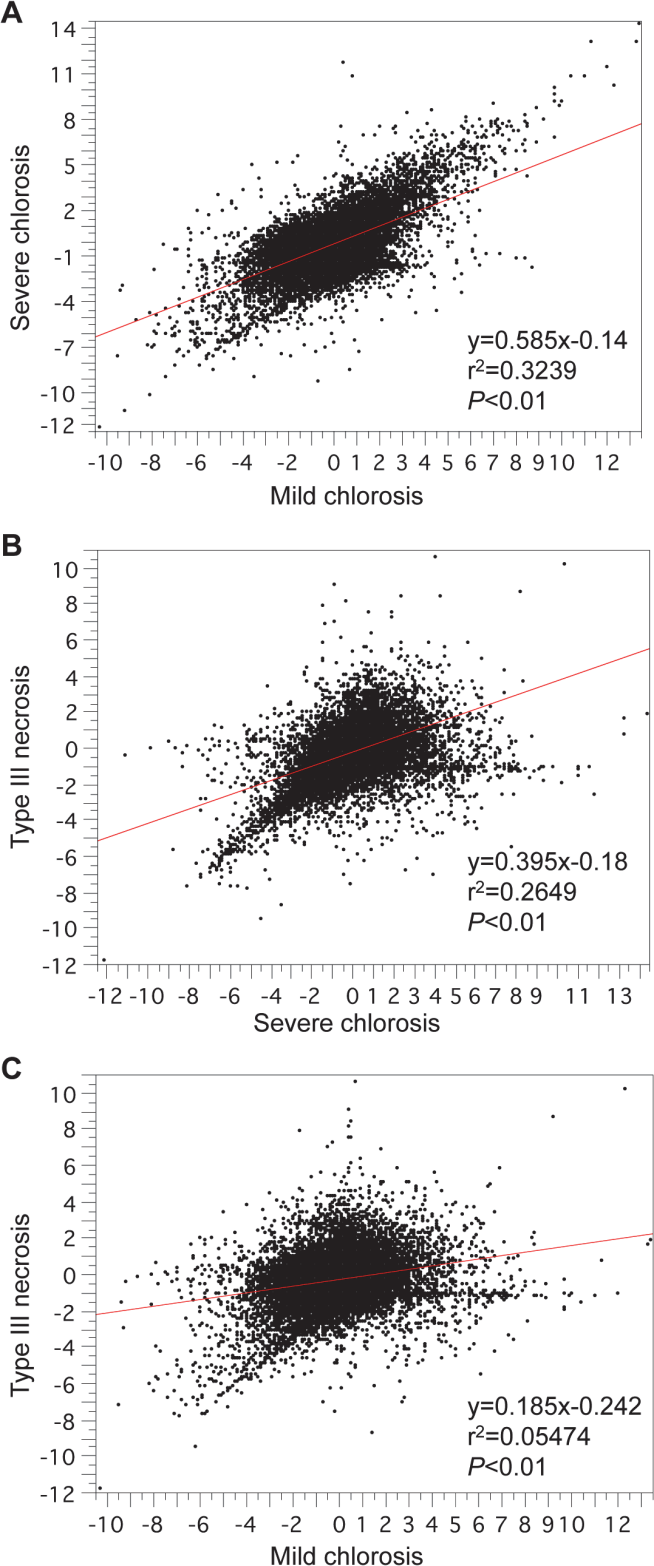
Correlation between the log_2_ ratios of genes with altered expression among plants with severe and mild chlorosis and type III necrosis. (A) Scatter diagram of the genes with altered expression in mild and severe chlorosis. (B) Scatter diagram of the genes with altered expression in severe chlorosis and type III necrosis. (C) Scatter diagram of the genes with altered expression in type III necrosis and mild chlorosis.

We compared gene expression profiles among the hybrid chlorosis lines and a type III necrosis line, which was a synthetic wheat line of Ldn/KU-2828 [11]. The signal-intensity differences of all probes relative to WT signals were compared among leaves showing mild and severe hybrid chlorosis and type III hybrid necrosis. Significantly strong positive correlations were detected between the mild and severe hybrid chlorosis lines and between the severe hybrid chlorosis and type III necrosis lines (Fig. 5A-B). However, no correlation of signal intensity was observed between leaves showing mild chlorosis and type III necrosis (Fig. 5C).

Moreover, the expression profiles of the severe and mild hybrid chlorosis and type III necrosis lines were compared using defense-, carbohydrate metabolism-, and photosynthesis-related probes in which expression levels were changed for each growth abnormality relative to WT (Table 5, S1–S3 Tables). Significant positive correlations were observed between the profiles of the defense-related genes in mild and severe chlorosis and between the profiles of these genes in hybrid chlorosis and type III necrosis. Similar observations were found in the expression profiles of carbohydrate metabolism-related genes between mild and severe chlorosis. In photosynthesis-related genes, however, significant positive correlations were detected in some of the combinations.

**Table 5:** Comparison of gene expression profiles among leaves of synthetic wheat with severe chlorosis, mild chlorosis and type III necrosis phenotypes.

Query	Number of probes	Target expression profile	Correlation coefficient
Defense-related genes up-regulated			
in severe chlorosis vs. WT	89	Mild chlorosis vs. WT	0.982**
in severe chlorosis vs. WT	89	Type III necrosis vs. WT^a^	0.970**
in mild chlorosis vs. WT	53	Severe chlorosis vs. WT	0.924**
in mild chlorosis vs. WT	53	Type III necrosis vs. WT^a^	0.834**
in type III necrosis vs. WT^a^	221	Severe chlorosis vs. WT	0.702**
in type III necrosis vs. WT^a^	221	Mild chlorosis vs. WT	0.496**
Carbohydrate metabolism-related genes up-regulated			
in severe chlorosis vs. WT	105	Mild chlorosis vs. WT	0.433**
in severe chlorosis vs. WT	105	Type III necrosis vs. WT^a^	0.677**
in mild chlorosis vs. WT	55	Severe chlorosis vs. WT	0.466**
in mild chlorosis vs. WT	55	Type III necrosis vs. WT^a^	-0.001
in type III necrosis vs. WT^a^	77	Severe chlorosis vs. WT	0.553**
in type III necrosis vs. WT^a^	77	Mild chlorosis vs. WT	0.374**
Photosynthesis-related genes down-regulated			
in severe chlorosis vs. WT	72	Mild chlorosis vs. WT	0.313**
in severe chlorosis vs. WT	72	Type III necrosis vs. WT^a^	0.329**
in mild chlorosis vs. WT	71	Severe chlorosis vs. WT	-0.278*
in mild chlorosis vs. WT	71	Type III necrosis vs. WT^a^	-0.345**
in type III necrosis vs. WT^a^	13	Severe chlorosis vs. WT	0.705*
in type III necrosis vs. WT^a^	13	Mild chlorosis vs. WT	0.608

The Pearson coefficient values were calculated based on the differences in signal intensities of three sets of the categorized probes, defense-related genes un-regulated, carbohydrate metabolism-related genes un-regulated and photosynthesis-related genes down-regulated, between the wild-type and abnormal growth hybrids.

Significant correlations

*, *P* < 0.05

**, *P* < 0.01

^a^Mizuno et al. [11]

### Expression analysis of senescence- and defense-related genes

**Fig 6:**
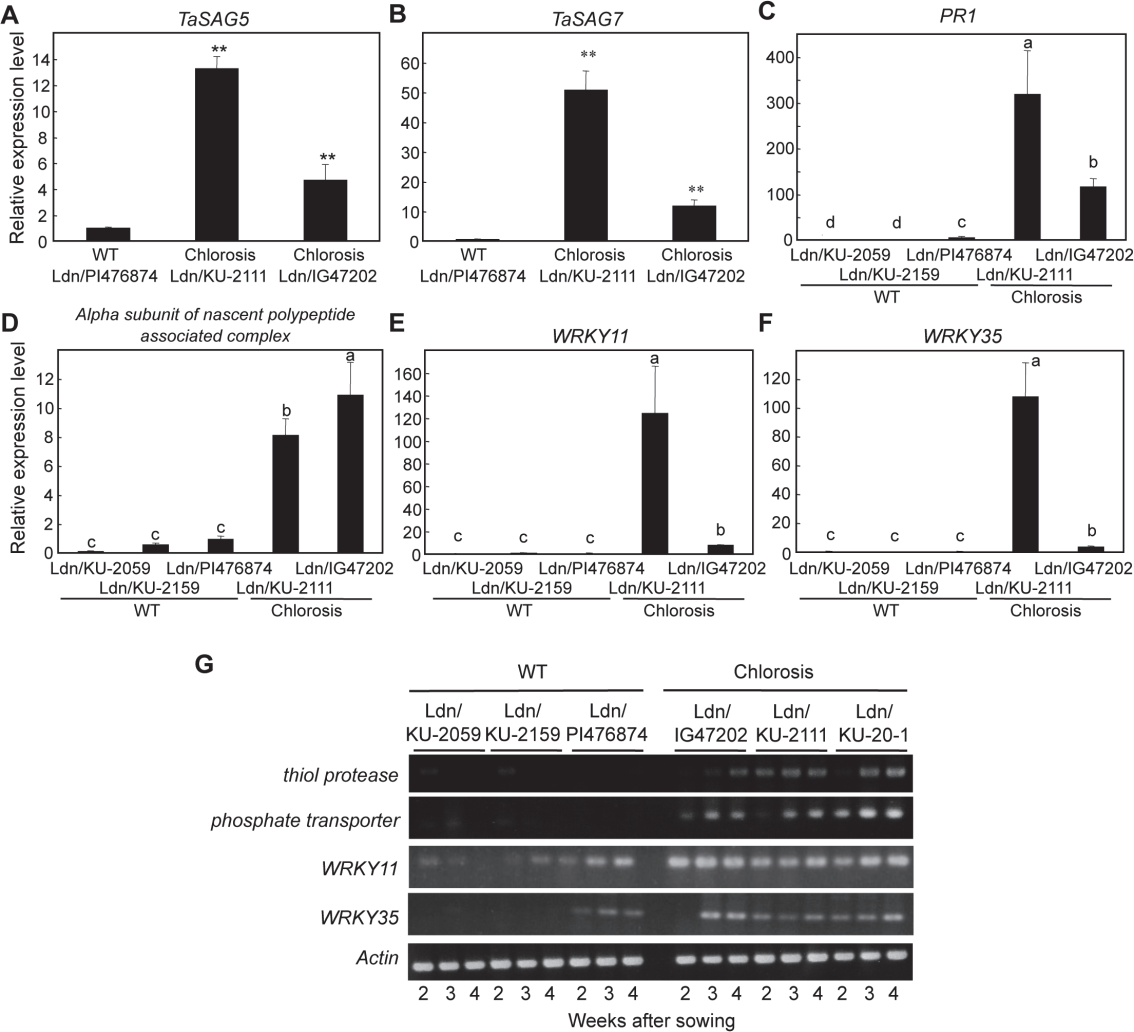
Transcript accumulation of the *SAGs* and other genes identified in microarray analysis, as revealed by RT-PCR and quantitative RT-PCR. (A,B) Two wheat *SAGs*. Student’s *t*-test was used for statistical significance (**P < 0.01) of line differences. Total RNA was extracted from the first leaves of the 3-week-old seedlings. Results represent mean ± standard errors (n = 3). The *Actin* gene was used as an internal control. (C-F) Four genes identified in microarray analysis. Mean values with the same letters were not significantly (*P* < 0.05) different (Tukey-Kramer’s HSD test). Results represent mean ± standard errors (n = 3). The *Actin* gene was used as an internal control. (G) RT-PCR analysis of four genes up-regulated in hybrid chlorosis.

To validate the microarray data, quantitative reverse transcription (RT)-PCR was conducted for six up-regulated genes. Two senescence-associated genes, *TaSAG5* and *TaSAG7* [37], were significantly up-regulated in leaves of the two hybrid chlorosis lines compared with the WT synthetic wheat line, and transcript levels were higher in the severe chlorosis line than in the mild chlorosis line (Fig. 6A-B). Similarly, a defense-related gene, *PR1*, was dramatically activated in hybrid chlorosis, and its transcript levels correlated well with the severity of chlorosis (Fig. 6C). Up-regulation of a gene encoding the alpha chain of nascent-polypeptide-associated complex, which can reversibly bind to ribosomes and regulate translocation of newly synthesized proteins [38,39], was observed in hybrid chlorosis (Fig. 6D), but not in type III necrosis [11]. Two WRKY transcription factor genes, *WRKY11* and *WRKY35*, were significantly up-regulated in hybrid chlorosis, and their transcript levels were higher in 3-week-old seedlings of the severe chlorosis line than the mild chlorosis line (Fig. 6E-F).

Next, RT-PCR analysis of four selected genes up-regulated in hybrid chlorosis was conducted using 2-, 3-, and 4-week-old seedling leaves of three WT and three hybrid chlorosis lines. This time-course study confirmed the results from microarrays and quantitative RT-PCR. Transcript accumulation of the four genes was more abundant in the three hybrid chlorosis lines than in the three WT lines of synthetic wheat (Fig. 6G).

### Comparison of photosynthetic activity among synthetic wheat lines

**Fig 7:**
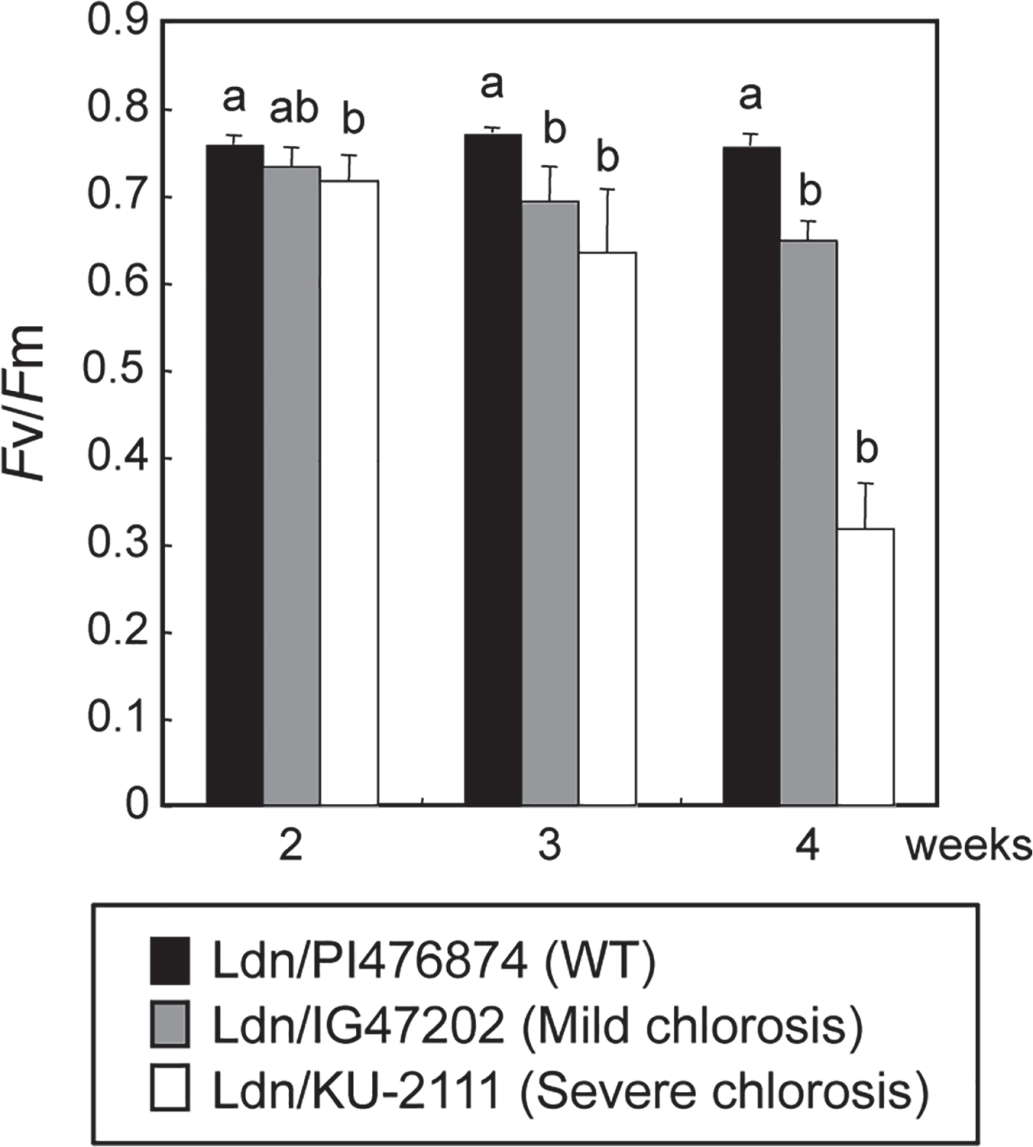
Comparison of photosynthetic activity of WT and chlorosis lines based on chlorophyll fluorescence. Each plant was grown at 23°C for 2 to 4 weeks. Mean values with the same letters were not significantly different (*P* < 0.05) (Tukey-Kramer’s HSD test).

To examine the effects of down-regulation of photosynthesis-related genes, photosynthetic activity was compared among three synthetic wheat lines, WT Ldn/PI476874 and hybrid necrotic lines Ldn/IG47202 and Ldn/KU-2111. The photosystem II activities of the chlorosis-exhibiting lines were significantly reduced in the first leaves of 3- and 4-week-old seedlings compared to those of the WT line (Fig. 7), which is consistent with the down-regulation of photosynthesis-related genes in hybrid chlorosis. The reduction of photosystem II activity appeared more remarkable in the severe chlorosis line than in the mild chlorosis line, and the *Fv*/*Fm* value was significantly different in severe chlorosis plants grown for 2 weeks.

### Estimation of agricultural traits in hybrid chlorosis lines

To assess the effects of hybrid chlorosis on various agricultural traits, we grew two F_2_ populations derived from crosses of WT to chlorosis synthetic wheat lines, Ldn/CGN10768//Ldn/IG47202 (*N* = 103) and Ldn/PI476874//Ldn/KU-2111 (*N* = 108). The former population was from a cross between the WT and mild chlorosis lines, and the latter between the WT and severe chlorosis lines. The parental synthetics contained the A and B genomes from Ldn and the diverse D genomes originating from the *Ae*. *tauschii* accessions. In each F_2_ population, the individuals showing chlorosis were distinguished from the WT individuals, and the 11 agricultural traits listed in Table 6 were compared.

**Table 6:** Comparison of agricultural characters in F_2_ individuals between WT and hybrid chlorosis lines.

Agricultural character	Ldn/CGN10768//Ldn/IG47202 (WT/mild chlorosis)		Ldn/PI476874//LdnKU-2111 (WT/severe chlorosis)	
	WT (*N* = 27)	chlorosis (*N* = 76)	WT (*N* = 33)	chlorosis (*N* = 75)
Column length (cm)	119.94 ± 16.9	112.52 ± 16.9	118.53 ± 21.5	95.59 ± 19.9**
Spike length (cm)	12.35 ± 1.04	12.01 ± 1.18	11.60 ± 1.82	10.85 ± 2.16
First internode length (cm)	47.17 ± 6.81	44.16 ± 9.21	45.67 ± 11.26	37.72 ± 10.14**
Second internode length (cm)	20.51 ± 3.43	19.69 ± 3.09	21.02 ± 4.50	17.19 ± 4.55**
Third internode length (cm)	17.56 ± 2.92	15.75 ± 2.72**	16.63 ± 3.57	13.34 ± 2.52**
Selfed seed fertility (%)	44.81 ± 25.85	35.12 ± 21.73	71.37 ± 18.92	45.88 ± 8.90**
Seed number/spike	14.04 ± 9.18	10.76 ± 7.10	23.08 ± 6.73	13.87 ± 10.8**
100-seeds weight	4.57 ± 0.74	4.20 ± 0.90	4.51 ± 1.10	3.02 ± 1.07**
Heading days	153.36 ± 2.58	154.44 ± 2.44	154.49 ± 3.51	157.81 ± 4.42**
Flowering days	159.80 ± 2.47	160.97 ± 3.12	161.34 ± 4.32	165.52 ± 5.68**
Maturation days	192.81 ± 2.05	194.17± 3.12*	194.32 ± 3.49	198.61 ± 3.65**

Means ± SD were calculated, and Student’s *t*-test was used to test for statistical significance (*, *P* < 0.05; **, *P* < 0.01) between the WT and chlorosis lines.

In the Ldn/PI476874//Ldn/KU-2111 population, significant differences between the WT and chlorosis F_2_ individuals were found in all examined traits except for spike length (Table 6). The severe chlorosis plants showed a phenotype of reduced height, low seed fertility, small seed weight, and late flowering compared with the WT plants. On the other hand, no significant differences were observed in most examined traits between the WT and mild chlorosis plants. Significant reductions in third internode length and delay of maturation time were detected in the mild chlorosis plants.

### Disease resistance in hybrid chlorosis lines

A number of defense-related genes were up-regulated in the hybrid chlorosis lines, as revealed by microarray analyses (Fig. 4). For evaluation of disease resistance to a *Triticum* isolate, Br48, of the blast fungus (*Magnaporthe oryzae*, syn. *Pyricularia oryzae*) [40], two WT synthetic wheat lines, Ldn/CGN10768 and Ldn/PI476874, and two showing chlorosis, Ldn/IG47202 and Ldn/KU-2111, were inoculated with Br48. As previously reported [41], green and brown infection types are respectively considered to be susceptible (or virulent) and resistant (or avirulent). In all examined lines, 1- and 2-week-old seedlings showed infection types 4G and 5G [41], meaning that these seedlings were susceptible to Br48 (Table 7). However, the infection types of 3-week-old seedlings were 1B, 2B, and 4B in both chlorosis lines but not in the WT synthetic lines. These observations clearly indicated that the hybrid chlorosis-showing plants were more resistant to the wheat blast fungus than the WT plants at the 3-week-old seedling stage.

**Table 7:** Responses of four synthetic wheat lines to the wheat blast fungus Br48.

Synthetic line	Phenotype	Age of seedlings	Temperature after inoculation	Infection type*	Putative disease resistance**
Ldn/CGN10768	WT	1-week-old	22°C	5G	S
			26°C	5G	S
		2-week-old	22°C	4G	S
			26°C	5G	S
		3-week-old	22°C	4G	S
			26°C	5G	S
Ldn/PI476874	WT	1-week-old	22°C	5G	S
			26°C	5G	S
		2-week-old	22°C	4G	S
			26°C	5G	S
		3-week-old	22°C	4G	S
			26°C	5G	S
Ldn/IG47202	Mild chlorosis	1-week-old	22°C	5G	S
			26°C	5G	S
		2-week-old	22°C	5G	S
			26°C	5G	S
		3-week-old	22°C	1B	R
			26°C	4B	R
Ldn/KU-2111	Severe chlorosis	1-week-old	22°C	5G	S
			26°C	5G	S
		2-week-old	22°C	4G	S
			26°C	5G	S
		3-week-old	22°C	2B	R
			26°C	4B	R

*Infection type with Br48 was estimated according to Chuma et al. [41].

**S; susceptible, R; resistant

**Fig 8:**
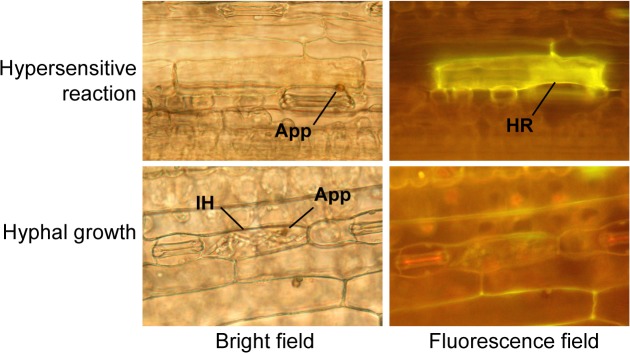
Hypersensitive reaction (upper) and hyphal growth (lower) in epidermal cells of synthetic hexaploid wheat lines 48 h after inoculation with Br48, observed under a bright field (left) or fluorescence field (right). Each plant was grown at 23°C for 1 to 3 weeks. App; appressoria, HR; hypersensitive reaction, IH; infection hyphae.

To examine the effects of hybrid chlorosis on disease resistance, we compared cellular responses to the wheat blast fungus in the first leaves of mild chlorosis and WT lines of synthetic wheat. The inoculated leaves were fixed 48 h after inoculation with Br48, and observed under a fluorescent microscope. Appressoria of Br48 formed on epidermal cells of the leaves (Fig. 8). In some attacked cells, the hypersensitive reaction (HR) occurred, which was accompanied by strong yellow fluorescence as previously observed [40]. In other cells with no fluorescence, no resistance reaction to the appressorial penetration was observed, and infection hyphae were well developed (Fig. 8).

In most attacked epidermal cells of the highly susceptible barley cultivar Nigrate, HR was rarely observed and the infection hyphae were successfully developed (Table 8). The WT and hybrid chlorosis lines of wheat synthetics showed partial resistance to Br48, and HR was observed in the first leaves of 1-, 2-, 3-week-old seedlings. HR was more frequently observed in 2- and 3-week-old seedlings of the mild chlorosis line than in those of the WT synthetic wheat line (Table 8). The HR frequency was dramatically increased from one to two weeks after seed sowing in the mild chlorosis line. The significant differences in cellular responses to Br48 infection indicated that seedlings of the mild chlorosis line are more resistant to Br48 than the WT.

**Table 8:** Cellular responses of the first leaves in mild chlorosis and WT lines to the wheat blast fungus Br48.

Weeks after sowing	Line	Percentage of germlings inducing	
		HR**	Hyphal growth
1	Ldn/IG47202 (Mild chlorosis)	33.79±11.71^a^	66.21±11.71^b^
	Ldn/CGN10768 (WT)	28.18±3.00^a^	71.82±13.52^b^
	Nigrate*	0.29±0.50^b^	99.71±4.10^a^
2	Ldn/IG47202 (Mild chlorosis)	61.06±13.52^a^	38.94±3.01^b^
	Ldn/CGN10768 (WT)	26.52±10.95^b^	73.48±10.95^a^
	Nigrate	3.66±1.56^b^	96.33±1.91^a^
3	Ldn/IG47202 (Mild chlorosis)	68.90±4.11^a^	31.10±0.50^c^
	Ldn/CGN10768 (WT)	16.03±1.91^b^	83.97±1.56^b^
	Nigrate	0.00±0.00^c^	100±0.00^a^

Mean values with the same letters were not significantly different (*P* < 0.05) (Tukey-Kramer’s HSD test).

*A barley cultivar Nigrate was used as a control.

**Hypersensitive reaction

Moreover, we examined the defense response to an isolate, Th-1, of the wheat powdery mildew fungus (*Blumeria graminis* f. sp. *tritici*) in the first leaves of mild chlorosis and WT lines of synthetic wheat. The WT synthetic wheat line Ldn/PI476874 showed clear powdery mildew resistance to Th-1 (S1 Fig.). Since Ldn was completely sensitive to Th-1, the resistance of Ldn/PI476874 would be transmitted from the parental *Ae*. *tauschii* accession PI476874 which revealed also clear resistance to Th-1. The mild chlorosis line Ldn/IG47202 showed the necrotic cell death on leaves. The area and number of the necrotic lesions decreased in the three-week-old plants of Ldn/IG47202 in which the yellow lesions started to appear. No necrotic lesion appeared on non-infected leaves in Ldn/IG47202, and thus the necrotic lesions were generated by the infection of Th-1. These observations suggested that the area and number of the necrotic lesions were negatively correlated with appearance of the yellow lesions in the mild chlorosis line of synthetic wheat.

## Discussion

Allopolyploidy provides advantages to establishment of new plant species, including fixation of heterosis [1]. However, hybrid growth abnormalities, observed frequently in interspecific crosses, inhibit allopolyploid speciation. Hybrid chlorosis, observed in some cross combinations between tetraploid wheat and *Ae*. *tauschii*, is recognized as one such growth abnormality in allohexaploid wheat speciation [11]. In hybrid chlorosis lines of synthetic allohexaploid wheat, yellowish lesions and sectors on the leaf surface appear gradually in older tissues. In type III necrosis, necrotic cell death also gradually begins from older tissues, whereas the lesion color is clearly different from that in hybrid chlorosis. In type III necrosis, necrotic cell death is triggered by an HR-like reaction and accompanied by generation of reactive oxygen species [11]. Within the chloroplasts of mesophyll cells in leaves undergoing type III necrosis, oil droplets, meaning plastoglobuli, are more frequently found than in leaves of WT. In addition, plasmolysis, collapse of vacuoles, and organelle degradation are observed as typical features of dead cells in type III necrosis. Unlike type III necrosis, in leaves developing chlorosis, abnormal chloroplasts were found within mesophyll cells (Fig. 3). Plastoglobuli appeared before the yellowish sectors formed on the leaf surfaces of the plants showing hybrid chlorosis. Shrinkage of the chloroplasts occurred, with appearance of many plastoglobuli. This ultrastructural modification of chloroplasts is compatible with the typical features of hybrid chlorosis in the wheat synthetics, which is different from the features of type III necrosis.

It was previously reported that an increased number of plastoglobuli accompanies thylakoid breakdown during senescence [32]. In addition, oxidative stress to the photosynthetic apparatus increases the number of plastoglobuli [32]. Thus, an increase in the number of plastoglobuli seems to be due to generation of reactive oxygen species in chloroplasts of leaves with type III necrosis [11]. In comparative transcriptome analyses, the gene expression profile of plants showing hybrid chlorosis, especially severe chlorosis, resembled that of type III necrosis (Fig. 5, Table 5). Up-regulation of defense- and carbohydrate metabolism-related genes was commonly observed in severe and mild hybrid chlorosis and type III necrosis, which suggested that these hybrid incompatibilities could at least partly share common mechanisms to induce their symptoms. The number of genes detected with defense-related probes showing expression up-regulated at least 3 times more than in WT was more abundant for type III necrosis than hybrid chlorosis, whereas the number of up-regulated probes related to carbohydrate metabolism was more abundant for hybrid chlorosis than type III necrosis (Tables 1, 2 and 5). In the hybrid chlorosis lines, senescence-associated genes and *NAM-B1* were greatly up-regulated. *NAM-B1*, encoding a NAC transcription factor, functions to control leaf senescence and is associated with grain protein, zinc, and iron contents [36]. The rice *NAM-B1* homologs also act as regulators of leaf senescence [39,42]. Therefore, these expression profiles showed that hybrid chlorosis resulting from interaction between the AB and D wheat genomes has unique features distinct from hybrid necrosis, and suggests that senescence might be accelerated in the hybrid chlorosis lines of wheat synthetics. Abnormal chloroplasts are accompanied by an increase in the number of plastoglobuli generated in green leaves of the hybrid chlorosis line, and chloroplast degradation was observed in the yellowish leaves (Fig. 3). These TEM observations also supported the idea that accelerated senescence occurs in leaves of the hybrid chlorosis lines.

Hybrid incompatibility between two diverging lineages is generally explained by interaction of at least two loci or alleles [12]. The hybrid chlorosis observed in some crosses between tetraploid wheat and *Ae*. *tauschii* appears to be due to an epistatic interaction of two loci from the AB and D wheat genomes, considered to be a Dobzhansky-Muller-type hybrid barrier [11]. Several complementary genes for chlorophyll abnormalities have been studied in wheat. The *Ch1* hybrid chlorosis gene has been assigned to chromosome 2A in tetraploid and hexaploid wheat, and *Ch2* to chromosome 3D in hexaploid wheat [23–25]. Hybrid chlorosis in some interspecific crosses of tetraploid wheat is respectively controlled by *Cs1* and *Cs2* on chromosomes 5A and 4G [22]. Three additional chlorophyll abnormalities are caused by the following complementary gene interactions: *sv1* (3A) and *sv2* (2A), *dv1* (2B) and *dv2* (supposedly 2A), and *abn1* (2A) and *abn2* (2B) in *T*. *turgidum* [26]. The D genome-located causal gene, *Hch1*, for hybrid chlorosis in ABD triploids and wheat synthetics was assigned to the short arm of chromosome 7D (Fig. 2). Thus, *Hch1* is a novel gene inducing hybrid chlorosis in *Triticum* species. We propose the presence of a gene, *Hch2*, in the AB genome of tetraploid wheat, which acts as a gene complementary to *Hch1* for hybrid chlorosis in ABD triploids. Although many complementary pairs of hybrid chlorosis genes have been reported, no locus has been successfully cloned in wheat. To understand the molecular basis of and physiological changes in hybrid chlorosis, map-based cloning of these causal genes would be required in future studies. In our previous study, a causal gene for type III necrosis in the D genome, *Nec1*, was also assigned to 7DS [11]. *Hch1* was located at a similar chromosomal region to *Nec1*. Because *Ae*. *tauschii* accessions inducing type III necrosis are genealogically distinct from those inducing hybrid chlorosis [11], the *Hch1* and *Nec1* alleles could have independently arisen in the *Ae*. *tauschii* population. Many disease resistance genes such as *Lr34* are concentrated around the *Hch1* and *Nec1* region of 7DS [30,43]. Most causal genes that have been isolated to date are related to disease resistance [13–16], which suggests that *Hch1* and *Nec1* may be any of the disease resistance genes located in the 7DS region.

In the hybrid chlorosis lines examined in the present study, a lot of WRKY transcription factor genes were up-regulated (Tables 3 and 4). The putative *Hch1*-*Hch2* interaction should activate multiple WRKY transcription factors in the hybrid chlorosis plants. WRKY transcription factors play important roles in biotic and abiotic stress responses and senescence in higher plants [34,35]. In particular, many *WRKY* genes have been identified to act as positive regulators of SA-dependent immune responses [44]. In rice, for example, *OsWRKY45* functions to regulate disease resistance against the rice blast fungus [45]. A number of defense-related genes, such as *PR1*, were also up-regulated in the hybrid chlorosis plants (Fig. 4), which could be at least partly controlled through activation of *WRKY* genes in these plants. Many *WRKY* genes are activated not only in hybrid chlorosis lines but also in wheat ABD triploids showing hybrid necrosis and severe growth abortion [11,19,20]. The direct targets of the epistatic interaction of causal genes for these types of hybrid incompatibility should be studied to clarify the signaling pathways for transcriptional activation of the *WRKY* genes in the abnormal ABD triploid plants.

Few significant differences in agricultural traits were found in WT synthetic wheat plants and those showing mild chlorosis, although remarkable growth inhibition was observed in plants showing severe chlorosis (Table 6). Transcripts of defense-related and *WRKY* genes accumulated abundantly in leaves of the plants showing mild chlorosis, which in fact showed enhanced resistance to the wheat blast fungus through HR (Tables 7 and 8). The enhanced levels of wheat blast disease resistance were correlated with progress of chlorosis symptoms (Tables 7 and 8). In addition, we confirmed disease resistance to wheat powdery mildew fungus. The WT synthetic wheat line used in the disease resistance assay unfortunately showed clear wheat powdery mildew resistance because the powdery mildew resistance gene was transmitted from the parental *Ae*. *tauschii* accession PI476874, whereas the powdery mildew resistance also appeared to be enhanced in the mild chlorosis line of synthetic wheat (S1 Fig.). Such enhanced disease resistance to wheat blast and powdery mildew fungi was visibly confirmed in three-week-old plants of the mild chlorosis line in which the yellow lesions started to appear. These observations strongly suggest that the level of disease resistance is greatly strengthened in the mild chlorosis plants through up-regulation of defense-related genes. Generally, hybrid necrosis extensively inhibits plant growth and dramatically reduces yield, even if SA-dependent immune responses are activated in plants showing these symptoms [11,14,18], which is an undesirable effect for crop breeding. Tight association of cell death with defense responses and processes involved in senescence has been observed in some cell death-related mutants of *Arabidopsis*. A loss-of-function mutant analysis of *ACCELERATED-CELL-DEATH11* (*ACD11*) in *Arabidopsis*, for example, implied that both defense- and senescence-related signal pathways may lead to programmed cell death in plant leaves [46]. A recent study of allelic variation at the *ACD6* locus in *Arabidopsis* revealed a negative correlation between the amount of vegetative growth and level of resistance to pathogens [47]. The hyperactive *ACD6* allele, inducing delayed growth and reduced biomass, strongly enhances disease resistance. This result indicates that some lesion-mimic mutant alleles can provide significant advantages for pathogen attack despite their negative effects on biomass. In the case of hybrid chlorosis triggered by the *Hch1*-*Hch2* interaction, a similar trade-off between disease resistance and reduced biomass could be assumed. In particular, it might be expected that the negative effects on biomass of mild chlorosis-showing plants could be minimized and substantial fitness conveyed under pathogen-polluted environments. Activation of SA-related immune responses surely reduces fitness in the absence of pathogens [48], but increases it in the presence of pathogens [49]. Fine-tuning of the trade-offs of the *Hch1*-*Hch2* interaction would be required for substantial use of mild chlorosis in wheat breeding under inferior environments, such as fields with frequent occurrence of disease. Production of near-isogenic lines for *Hch1* and *Hch2* should be effective for precise evaluation of the trade-offs between disease resistance and reduced biomass in further studies.

## Materials and Methods

### Plant materials

In our previous study, a tetraploid wheat accession Ldn was used as the female parent, and crossed with 122 *Ae*. *tauschii* accessions to artificially produce triploid wheat hybrids [11]. Selfed seeds (F_2_ generation), called synthetic wheats, from the triploid F_1_ hybrids were obtained through unreduced gamete formation [8,50]. In this study, we used ten synthetic hexaploid wheat lines (F_3_ generation) derived from ten cross combinations between Ldn and ten *Ae*. *tauschii* accessions, PI476874, KU-2059, KU-2078, KU-2126, KU-2159, CGN10768, KU-20-1, KU-2111, IG47202 and KU-2069 [9,11]. The six synthetic lines, Ldn/PI476874, Ldn/KU-2059, Ldn/KU-2078, Ldn/KU-2126, Ldn/KU2159 and Ldn/CGN10768, showed a normal growth phenotype (WT), and the Ldn/KU-20-1, Ldn/KU-2111 and Ldn/IG47202 synthetic wheat lines exhibited hybrid chlorosis. The chlorosis phenotypes of Ldn/KU-2111 and Ldn/KU-20-1 were more severe than that of Ldn/IG47202. The Ldn/KU-2069 synthetic line was used as a type III hybrid necrosis line [11]. The synthetic wheat lines were grown individually in pots in the field at Kobe University to determine their growth phenotype.

### Mapping of the D-genome causal gene for hybrid chlorosis

For the mapping population, 96 F_2_ plants from a cross between WT (synthetic hexaploid wheat line Ldn/PI476874) and the severe hybrid chlorosis line (synthetic hexaploid wheat line Ldn/KU-2111) were used. Total DNA was extracted from leaves of the parental strains and F_2_ individuals, and SSR genotyping was performed. For SSR genotyping, 40 cycles of PCR were performed using 2x Quick Taq HS DyeMix (TOYOBO, Osaka, Japan) and the following conditions: 10 s at 94°C, 30 s at the annealing temperature, and 30 s at 68°C. Information on the SSR markers and their annealing temperature was obtained from the National BioResource Project (NBRP) KOMUGI web site (http://www.shigen.nig.ac.jp/wheat/komugi/strains/aboutNbrpMarker.jsp) and the GrainGenes web site (http://wheat.pw.usda.gov/GG2/maps.shtml). The PCR products were separated in 2% agarose or 13% non-denaturing polyacrylamide gels and visualized under UV light after staining with ethidium bromide. Genetic mapping was performed using the MAPMAKER/EXP version 3.0b package, and the threshold for log-likelihood scores was set at 3.0; genetic distances were calculated with the Kosambi function [51].

For mapping of leaf rust-resistance locus *Lr34* [43], the primer set 5’-TGCGGCGATTCTATACTACT-3’ and 5’-CCGACATCAAGAACCTCC-3’ was designed. The PCR-amplified products were digested by the 4-bp cutting restriction enzyme *Taq*I, and the digests were separated in a 13% non-denaturing polyacrylamide gel. In addition, seven markers were newly developed (S4 Table). Three SSR markers were designed based on survey sequence data for the short arm of chromosome 7D [30], searching for SSR motifs using SciRoKo version 3.4 software [52]. Additional SNPs were identified in target gene regions by sequencing approximately 700 bp of amplified DNA of *Ae*. *tauschii* accessions PI476874 and KU-2111. The target gene regions were selected based on reference sequences of *Brachypodium* and RNAseq data for *Ae*. *tauschii* leaves [31,53]. The nucleotide sequences were determined using an Applied Biosystems 3730*xl* DNA Analyzer (Applied Biosystems, Foster City, CA, USA), and SNPs were found through sequence alignment using GENETYX-MAC version 12.00 software (Whitehead Institute for Biomedical Research, Cambridge, MA, USA). The identified SNPs were converted to CAPS markers. For these CAPS markers, the PCR annealing temperatures were 54 or 58°C. The PCR fragments digested by the indicated restriction enzymes were separated in 13% non-denaturing polyacrylamide gels and stained with ethidium bromide (S4 Table).

### TEM observation

Seedling leaves of two synthetic hexaploid wheat lines, Ldn/PI476874 for WT and Ldn/KU-2111 for severe chlorosis, grown at 23°C for 3 weeks, were used for TEM. The leaf blades were cut into 1 mm^2^ pieces and then incubated in a freshly prepared solution of 5 mM CeCl_3_ buffered with 50 mM 3-(N-morpholino)propanesulfonic acid at pH 7.2 for 1 h. The fixation of samples and the sectioning of ultrathin sections 90 nm thick were conducted according to our previous report [20]. Sections were examined with a Hitachi-7100 electron microscope (Hitachi, Tokyo, Japan) at an accelerating voltage of 75 kV. Three sections cut from leaf resin blocks of two plants were used for ultrastructural analysis of chloroplasts. More than 25 mesophyll cells were examined in each of the sections. The areas of three different structures, such as chloroplasts, grana and plastoglobuli, were measured by Image J software (National Institutes of Health, USA; http://imagej.nih.gov/ij/).

### Transcriptome analysis

Three synthetic hexaploid wheat lines, Ldn/PI476874 as WT, Ldn/KU-2111 with severe hybrid chlorosis and Ldn/IG47202 with mild hybrid chlorosis, were used for microarray analysis. Total RNA was extracted from leaves of WT and hybrid chlorosis lines grown at 23°C for 3 weeks using an RNeasy Plant Mini kit (Qiagen, Hilden, Germany). A KOMUGI 38k oligonucleotide DNA microarray (Agilent Technologies, Santa Clara, CA) was supplied by the National BioResource Project (NBRP)-Wheat, Japan (https://www.nbrp.jp) for analysis. Detailed information on the 38k microarray platform can be found in Kawaura et al. [33] and the Gene Expression Omnibus (GEO) database of the National Center for Biotechnology Information (NCBI) website under GPL9805. Hybridization of Cy3-labeled cRNA, washing and image scanning were performed according to our previous report [20]. Two independent experiments were conducted for each sample. All microarray data were deposited as GSE59640 in the NCBI GEO database (http://www.ncbi.nlm.nih.gov/geo/), including supplementary files, GSM1441345-1441350.

The functions of probes and genes were predicted by both BLAST and BLASTx searches (*E* value < 1e^-10^) against the DNA Data Bank of Japan (DDBJ) database. The NBRP KOMUGI website (http://www.shigen.nig.ac.jp/wheat/komugi/array/index.jsp) was also used for functional identification of the proteins encoded by the probes. The UniProtKB Protein Knowledgebase (http://www.uniprot.org) was used for functional categorization of identified genes.

### RT-PCR and quantitative RT-PCR analyses

RT-PCR and quantitative RT-PCR were performed using RNA isolated from leaves of the three WT (Ldn/PI476874, Ldn/KU-2059, and Ldn/KU-2159) and three chlorosis (Ldn/IG47202, Ldn/KU-2111, and Ldn/KU-20-1) lines. Each plant was grown at 23°C for 3 weeks, and total RNA was extracted using Sepasol-RNA I (Nacalai Tesque, Kyoto, Japan). First-strand cDNA was synthesized from DNase I-treated RNA samples using ReverTra Ace Reverse Transcriptase (Toyobo, Osaka, Japan) and an oligo(dT)_20_ primer. The gene-specific primer sets for RT-PCR and qRT-PCR are listed in S5 Table. PCR-amplified products were separated by electrophoresis in a 1.5% agarose gel and stained with ethidium bromide. The transcript accumulation of each gene was detected by quantitative RT-PCR using a LightCycler 480 Real-Time PCR System (Roche Diagnostics, Mannheim, Germany) with THUNDERBIRD SYBR qPCR Mix (Toyobo) and gene-specific primer sets. The *Actin* gene was used as an internal control, and relative expression was calculated as 2^-ΔΔCt^, representing the value relative to the transcript levels in leaves of WT (Ldn/PI476874).

### Photosynthetic activity

A portable JUNIOR-PAM fluorometer (Heinz Walz GmbH, Effeltrich, Germany) was used for measurement of the maximum photochemical quantum yield of photosystem II (*Fv/Fm*). The first leaves of 2-, 3- and 4-week-old WT and hybrid chlorosis plants were incubated under dark conditions for 1 h and *Fv/Fm* was measured at ten independent locations on each leaf blade according to the instructions in the operating manual for the fluorometer.

### Measurement of agricultural traits

Seeds of F_2_ individuals from two crosses of the synthetic hexaploid lines, between Ldn/CGN10768 (WT) and Ldn/IG47202 (mild chlorosis) and between Ldn/PI476874 (WT) and Ldn/KU-2111 (severe chlorosis), were sown in November 2010, and plants were grown individually in pots arranged randomly. Eleven traits, column and spike length, length of the first, second and third internodes, selfed seed fertility, seed number per spike, hundred grain weight, and days to heading, flowering and maturation, were measured on the earliest tiller of each F_2_ plant.

### Evaluation of disease resistance

A field isolate, Br48, of the wheat blast fungus was used to assay disease resistance. Br48 is virulent on most accessions of hexaploid and tetraploid wheat [41]. Twelve seeds of each line were sown, and the adaxial surface of leaves of 1-, 2- and 3-week-old seedlings was sprayed with a conidial suspension (1–2 x 10^5^ conidia/mL) of Br48. The preparation of the suspension and incubation of inoculated plants in humid boxes were performed according to Chuma et al. [41]. The inoculated plants were then transferred to growth chambers at 22°C and 26°C, and disease symptoms were rated 5 d after inoculation. The size and rate of disease symptoms were evaluated according to Chuma et al. [41]. The size was rated using six progressive grades from 0 to 5, and the disease lesions were classified into two categories on the basis of their color, brown (B) or green (G). The infection types were represented as the combination of the size and color [41]. In addition, 48 h after inoculation at 22°C, the inoculated leaves were harvested and fixed for microscopic observation according to Murakami et al. [40]. HR and hyphal growth were evaluated under bright and fluorescent fields of a fluorescence microscope with excitation filter B (Olympus, Tokyo, Japan). The barley cultivar Nigrate, used as a control, is highly susceptible to Br48 [54].

An isolate, Th-1, of wheat powdery mildew fungus was also used to assay disease resistance. For preparation of inoculum, 10-d-old seedlings of a wheat cultivar ‘Norin 4’ were inoculated with conidia of Th-1 and maintained in growth chambers at 22°C. Twelve seeds of each line were sown, and the adaxial surface of leaves of 1-, 2- and 3-week-old seedlings was inoculated with Th-1 by blowing conidia from the infected seedlings of Norin 4. The inoculated plants were then transferred to the growth chambers, and disease symptoms were observed 8 d after inoculation. The entire experiments for the powdery mildew resistance assay were repeated two times.

## Supporting Information

S1 Fig Responses of two synthetic wheat lines and their parental accessions to the wheat powdery mildew fungus. (PDF)

S1 Table Comparison of signal intensities of the top 20 up-regulated defense-related genes in leaves of the mild chlorosis line with those of severe chlorosis and type III necrosis lines. (PDF)

S2 Table Comparison of signal intensities of the top 20 up-regulated carbohydrate metabolism genes in leaves of the mild chlorosis line with those of severe chlorosis and type III necrosis lines. (PDF)

S3 Table Comparison of signal intensities of the top 20 down-regulated photosynthesis-related genes in leaves of the mild chlorosis line with those of severe chlorosis and type III necrosis lines. (PDF)

S4 Table Primer sets used in *Hch1* mapping. (PDF)

S5 Table Primer sets used in quantitative RT-PCR. (PDF)
