# Supplementary material for: Accelerated Senescence and Enhanced Disease Resistance in Hybrid Chlorosis Lines Derived from Interspecific Crosses between Tetraploid Wheat and Aegilops tauschii
Source: PLoS One. 2015 Mar 25;10(3):e0121583. doi: 10.1371/journal.pone.0121583 (PMC4373817; doi:10.1371/journal.pone.0121583)
Supplement: S1 Fig — (PDF) [file pone.0121583.s001.pdf]

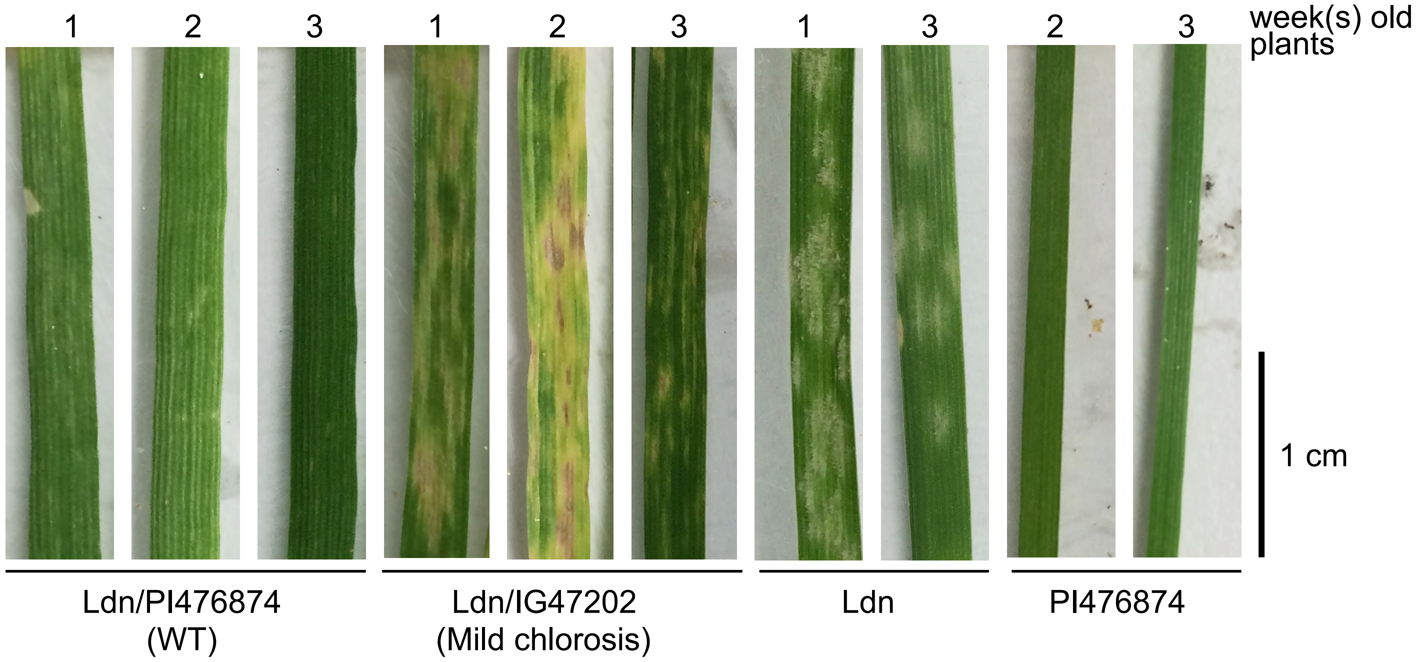

S1 Fig. Responses of two synthetic wheat lines and their parental accessions to the wheat powdery mildew fungus. Each plant was grown at 22°C for 1 to 3 weeks.
