# Supplementary material for: Accelerated Senescence and Enhanced Disease Resistance in Hybrid Chlorosis Lines Derived from Interspecific Crosses between Tetraploid Wheat and Aegilops tauschii
Source: PLoS One. 2015 Mar 25;10(3):e0121583. doi: 10.1371/journal.pone.0121583 (PMC4373817; doi:10.1371/journal.pone.0121583)
Supplement: S1 Table — (PDF) [file pone.0121583.s002.pdf]

**S1 Table. Comparison of signal intensities of the top 20 up-regulated defense-related genes in leaves of the mild chlorosis line with those of severe chlorosis and type III necrosis lines.**

| Probe name | Annotation | Ratio of mild chrolosis to WT | Ratio of severe chrolosis to WT | Ratio of type III necrosis to WT |
|---|---|---|---|---|
| ncbi_gi_1323745_269 | sulfur-rich/thionin-like protein | 10225.59 | 20803.66 | 1317.12 |
| whsh_allContig982 | chitinase (Chi2) | 4936.52 | 1257.91 | 1989.57 |
| wheat0130Contig6216 | chitinase 3 | 159.45 | 136.05 | 112.02 |
| rwhsh11j09 | Haem peroxidase, plant/fungal/bacterial family protein | 87.75 | 75.46 | 46.96 |
| wheat0130Contig10974 | pathogenesis-related protein 1 | 67.26 | 210.04 | 102.12 |
| MUGEST2003_23lib_Contig19505_653 | Atlas PR17d precursor | 56.75 | 16.22 | 17.63 |
| MUGEST2003_23lib_Contig8431_1180 | hypersensitive-induced response protein | 56.23 | 96.16 | 206.07 |
| wheat0130Contig7181 | WIR1, pathogen defense protein | 54.11 | 44.81 | 9.21 |
| rwhr18a08 | peroxidase 63 precursor | 49.93 | 23.70 | 6.34 |
| wheat0130Contig9475 | d6 pathogenesis-related protein 6 (pr6) | 49.73 | 81.71 | 371.41 |
| MUGEST2003_23lib_Contig5123_104 | peroxidase 1 precursor | 48.06 | 41.61 | 32.70 |
| MUGEST2003_23lib_Contig14776_137 | disease resistance response protein | 39.74 | 0.45 | 0.98 |
| wheat0130Contig5942 | chi gene for endochitinase | 35.21 | 63.18 | 28.88 |
| whh10h15 | Plant disease resistance response protein | 31.98 | 0.78 | 0.68 |
| wheat0130Contig9215 | wPR4a | 31.03 | 45.35 | 30.74 |
| rwhe8d06 | lipase class 3 family protein | 30.93 | 2.86 | 3.12 |
| wheat0130Contig2348 | PR-1.1 protein | 26.59 | 29.89 | 28.97 |
| whsh_allContig784 | iron/ascorbate-dependent oxidoreductase | 23.32 | 38.14 | 1.53 |
| wheat0130Contig5066 | Flavonol 4'-sulfotransferase | 21.40 | 53.95 | 14.19 |
| MUGEST2003_23lib_Contig18595_1325 | CYP72C-TA cytochrome P450 | 21.28 | 17.23 | 11.36 |
