# Supplementary material for: Accelerated Senescence and Enhanced Disease Resistance in Hybrid Chlorosis Lines Derived from Interspecific Crosses between Tetraploid Wheat and Aegilops tauschii
Source: PLoS One. 2015 Mar 25;10(3):e0121583. doi: 10.1371/journal.pone.0121583 (PMC4373817; doi:10.1371/journal.pone.0121583)
Supplement: S2 Table — (PDF) [file pone.0121583.s003.pdf]

S2 Table. Comparison of signal intensities of the top 20 up-regulated carbohydrate metabolism genes in leaves of the mild chlorosis line with those of severe chlorosis and type III necrosis lines.

Probe name	Annotation	Ratio of mild chrolosis to WT	Ratio of severe chrolosis to WT	Ratio of type III necrosis to WT
wheat0130Contig1783	beta-glucosidase	471.03	100.92	0.95
MUGEST2003_23lib_Contig16560_1036	beta-1,3-glucanase precursor (Glb3)	154.00	186.05	174.97
wheat0130Contig11052	putative cellulose synthase catalytic subunit (CesA8)	113.88	47.78	17.65
wheat0130Contig4161	CHS gene for chalcone synthase	101.18	31.31	270.62
MUG005D02F990129	TaGlu1a	93.81	29.02	4.38
wheat0130Contig13481	TaGlu1a	81.48	35.52	6.78
MUGEST2003_23lib_Contig16264_863	glucan endo-1,3-beta-D-glucosidase	78.36	116.16	35.98
MUG016C07R990620	TaGlu1a	76.67	31.99	6.74
wheat0130Contig13724	TaGlu1a	73.63	1.47	11.90
rwhyf23f20	trehalose-6-phosphate phosphatase	57.88	46.64	25.26
wheat0130Contig12804	CHS gene for chalcone synthase	52.22	23.06	190.45
rwhr9b21	cellulose synthase-like CslF3 (CslF3)	46.28	9.88	22.04
MUGEST2003_23lib_Contig3082_900	cellulose synthase-like CslF10	35.24	12.13	8.84
whkp10o03_288	acid beta-fructofuranosidase precursor	25.04	67.62	37.48
MUGEST2003_23lib_Contig22302_643	chalcone--flavonone isomerase	24.22	7.95	34.03
wheat0130Contig15644	sucrose:fructan 6-fructosyltransferase	21.99	104.93	478.22
wheat0130Contig4144	Terpene synthase-like domain containing protein	20.84	16.44	4.37
whfl_allContig771	Phosphatidylinositol N-acetylglucosaminyltransferase family protein	20.30	4.78	1.10
wheat0130Contig29	glucosyltransferase (GbssI)	19.36	0.58	1.01
wheat0130Contig5655	soluble acid invertase	19.32	100.80	36.09
