# Supplementary material for: Accelerated Senescence and Enhanced Disease Resistance in Hybrid Chlorosis Lines Derived from Interspecific Crosses between Tetraploid Wheat and Aegilops tauschii
Source: PLoS One. 2015 Mar 25;10(3):e0121583. doi: 10.1371/journal.pone.0121583 (PMC4373817; doi:10.1371/journal.pone.0121583)
Supplement: S3 Table — (PDF) [file pone.0121583.s004.pdf]

S3 Table. Comparison of signal intensities of the top 20 down-regulated photosynthesis-related genes in leaves of the mild chlorosis line with those of severe chlorosis and type III necrosis lines.

Probe name	Annotation	Ratio of mild chrolosis to WT	Ratio of severe chrolosis to WT	Ratio of type III necrosis to WT
whsh_allContig1173	PSII 10kD protein	0.0014	0.12	0.05
whsh_allContig984	PSII 10kD protein	0.0016	0.14	0.04
whsh_allContig1171	chlorophyll a/b-binding protein WCAB precursor	0.01	4.09	14.13
whsh_allContig1176	chlorophyll a/b-binding protein WCAB precursor	0.03	8.92	21.94
rwhv3n18a16_240	chlorophyll a/b-binding protein WCAB precursor	0.03	4.43	3.54
whsh_allContig905	chlorophyll a/b-binding protein WCAB	0.03	4.12	10.55
wheat0130Contig15419	Cab-phe4 chloroplast chlorophyll a/b binding protein	0.04	5.67	6.80
whsh_allContig113	chloroplast light-harvesting chlorophyll a/b binding protein	0.05	4.86	4.77
wheat0130Contig580	major chlorophyll a/b-binding protein	0.05	1.38	4.56
wheat0130Contig1365	chlorophyll a/b-binding protein WCAB precursor	0.06	10.61	24.74
whh2j01	chlorophyll a-b binding protein 2	0.07	0.22	0.29
whvh4b04_320	chloroplast envelope calcium ATPase precursor	0.08	0.58	0.30
wheat0130Contig16263	chloroplast DNA	0.08	0.29	1.44
whsh_allContig1263	chlorophyll a/b-binding preprotein	0.08	0.19	0.41
whsh_allContig1247	type I light-harvesting chlorophyll a/b-binding protein	0.09	0.18	0.46
whsh_allContig1037	chlorophyll a/b-binding preprotein	0.10	18.10	27.78
rwhsh14j20	major chlorophyll a/b-binding protein gene	0.10	7.81	16.69
wheat0130Contig7011	chlorophyll a/b-binding protein WCAB precursor (Wcab)	0.11	10.92	15.28
wheat0130Contig11783	major chlorophyll a/b-binding protein	0.11	1.90	3.53
rwhdl4i13	chlorophyll a/b-binding protein WCAB	0.14	3.88	9.89
