# Supplementary material for: Accelerated Senescence and Enhanced Disease Resistance in Hybrid Chlorosis Lines Derived from Interspecific Crosses between Tetraploid Wheat and Aegilops tauschii
Source: PLoS One. 2015 Mar 25;10(3):e0121583. doi: 10.1371/journal.pone.0121583 (PMC4373817; doi:10.1371/journal.pone.0121583)
Supplement: S4 Table — (PDF) [file pone.0121583.s005.pdf]

S4 Table. List of primer sets used in the *Hch1* mapping.

Locus	Forward and reverse primer sequences	Marker type	Restriction enzyme
Xkupg4	5'-AGGAGCTTCCTGGCTCTCAC-3'<br>5'-CGCGGCTCTTGTTGATTTTA-3'	SSR	
Xkupg12	5'-TCAAAAATTGGATCCCCAGA-3'<br>5'-ATGTGCCAGAGCCATGAGTT-3'	SSR	
Xkupg28	5'-GAAAGAAAGAAATAAGCAGAAAGAAAA-3'<br>5'-AGCGGTGAATCCTATTTGC-3'	SSR	
Bradi1g49880_1-3	5'-TGCAAAACTGGCAGAGAAGA-3'<br>5'-CCCCACTTTGAACATGGAAT-3'	CAPS	*Hae*III
Bradi1g48430_1-1	5'-CATGTTGCCATCACCTTCC-3'<br>5'-AAGCTACACAATATTTTGGATTTCA-3'	CAPS	*Apo*I
Bradi1g50050_1-4	5'-CTGCTGCGCCATTCTATTC-3'<br>5'-TAGAATGCAAGGGTGGCAAT-3'	CAPS	*Hha*I
*Xctg03994**	5'-GGCCTTTGTTTCATCAGCAT-3'<br>5'-GGGCAGCATGGTAGAAGTTG-3'	CAPS	*Afa*I

*Iehisa et al. [31]
