# Supplementary material for: Accelerated Senescence and Enhanced Disease Resistance in Hybrid Chlorosis Lines Derived from Interspecific Crosses between Tetraploid Wheat and Aegilops tauschii
Source: PLoS One. 2015 Mar 25;10(3):e0121583. doi: 10.1371/journal.pone.0121583 (PMC4373817; doi:10.1371/journal.pone.0121583)
Supplement: S5 Table — (PDF) [file pone.0121583.s006.pdf]

S5 Table. List of primer sets used in the RT-PCR and quantitative RT-PCR analyses.

Gene	Forward primer (5’ – 3’)	Reverse primer (5’ – 3’)
Actin	GCCGTGCTTTCCCTCTATG	GCTTCTCCTTGATGTCCCTTA
TaSAG5	GGVAAGGGGATGAGAATAG	CTTCTGATGCCTTCTTTGT
TaSAG7	CAAGCGCCCCTACACCGTCC	TGGTACTGCTGGGCGAAGAA
WRKY11	GGCGACGGCTCCTGCTT	TCCGCACAGCCATTATTTCC
WRKY35	GGAAATACGGGCAGAAAGAAA	GGGGCAAAACTGACGAGG
thiol protease	CTAATCCTTGTGGTCACGCT	CCCAGAGGTGTACGAGAG
phosphate transporter	CAGGACCCGCACAAGCG	ACATCTCCTCCAGCGACTTCC
Pathogenesis-related protein 1 (*PR1*)	ACGCCAACCAGAGGATCA	GAAGACGCCGAGGTTATT
Nascent polypeptide-associated complex	GGGAGGTGATGCTAGTGGAA	ACGGTCTTGCTCTGCTTGAT
